# Intrauterine exposure to chronic hypoxia in the rat leads to progressive diastolic function and increased aortic stiffness from early postnatal developmental stages

**DOI:** 10.14814/phy2.14327

**Published:** 2020-01-21

**Authors:** Praveen Kumar, Jude S. Morton, Amin Shah, Victor Do, Consolato Sergi, Jesus Serrano‐Lomelin, Sandra T. Davidge, Donna Beker, Jody Levasseur, Lisa K. Hornberger

**Affiliations:** ^1^ Division of Cardiology Department of Pediatrics University of Alberta Edmonton AB Canada; ^2^ Women and Children’s Health Research Institute University of Alberta Edmonton AB Canada; ^3^ Department of Obstetrics/Gynecology University of Alberta Edmonton AB Canada; ^4^ Cardiovascular Research Institute and Mazankowski Alberta Heart Institute University of Alberta Edmonton AB Canada; ^5^ Department of Laboratory Medicine and Pathology University of Alberta Edmonton AB Canada

**Keywords:** cardiovascular programming, development, diastolic dysfunction, echocardiography, intrauterine growth restriction (IUGR), myocardial function

## Abstract

**Aim:**

We sought to explore whether fetal hypoxia exposure, an insult of placental insufficiency, is associated with left ventricular dysfunction and increased aortic stiffness at early postnatal ages.

**Methods:**

Pregnant Sprague Dawley rats were exposed to hypoxic conditions (11.5% FiO_2_) from embryonic day E15‐21 or normoxic conditions (controls). After delivery, left ventricular function and aortic pulse wave velocity (measure of aortic stiffness) were assessed longitudinally by echocardiography from day 1 through week 8. A mixed ANOVA with repeated measures was performed to compare findings between groups across time. Myocardial hematoxylin and eosin and picro‐sirius staining were performed to evaluate myocyte nuclear shape and collagen fiber characteristics, respectively.

**Results:**

Systolic function parameters transiently increased following hypoxia exposure primarily at week 2 (*p* < .008). In contrast, diastolic dysfunction progressed following fetal hypoxia exposure beginning weeks 1–2 with lower early inflow Doppler velocities, and less of an increase in early to late inflow velocity ratios and annular and septal E’/A’ tissue velocities compared to controls (*p* < .008). As further evidence of altered diastolic function, isovolumetric relaxation time was significantly shorter relative to the cardiac cycle following hypoxia exposure from week 1 onward (*p* < .008). Aortic stiffness was greater following hypoxia from day 1 through week 8 (*p* < .008, except week 4). Hypoxia exposure was also associated with altered nuclear shape at week 2 and increased collagen fiber thickness at week 4.

**Conclusion:**

Chronic fetal hypoxia is associated with progressive LV diastolic dysfunction, which corresponds with changes in nuclear shape and collagen fiber thickness, and increased aortic stiffness from early postnatal stages.

## Graphical abstract

In the this study, the impact of chronic fetal hypoxia exposure on myocardial function and aortic stiffness was explored at early postnatal developmental stages using a previously described fetal hypoxia rat model. Fetal hypoxia exposure was associated with progressive LV diastolic dysfunction from 1‐2weeks, which evolved concomitant with changes in nuclear shape and collagen fiber thickness, and with increased aortic stiffness from day 1. These findings are likely relevant to long‐term cardiovascular disease observed following fetal growth restriction.

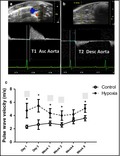

## 1 INTRODUCTION

Over 30% of all global deaths result from cardiovascular disease (CVD), including ischemic heart disease and systemic hypertension (World Health Organization, 1999). The American Heart Association has estimated that in the United States alone, 22.6% of the population has cardiovascular disease and the annual cost for treatment is $1 billion (National heart, lung, & blood institute, 2003). While many established risk factors for cardiovascular disease represent poor lifestyle choices in adulthood, it has become increasingly recognized that some risk factors or precursors develop even before birth as a consequence of exposure to an abnormal intrauterine environment (Barker, Winter, Osmond, Margetts, & Simmonds, 1989). This prenatal exposure may also contribute significantly to the growing trends in obesity, (Stocker, Arch, & Cawthorne, 2005) diabetes, (Stocker et al., 2005) and hypertension (Ojeda, Grigore, & Alexander, 2008) observed in the pediatric population (Kaijser et al., 2008).

The original observations of Dr Barker, who observed high rates of ischemic heart disease among adults of low birth weight, resulted in the development of the hypothesis that fetal adaptations to an abnormal in utero environment lead to permanent structural and functional differences in key organ systems, predisposing them to adult cardiovascular and metabolic disease (Barker et al., 1989). Previous investigations have demonstrated that intrauterine growth restriction (IUGR) is associated with long‐term adult CVD. In one large population study in Sweden, a birth weight of <2 standard deviations below the mean for gestational age was associated with an overall hazard ratio of 1.64 (95% confidence interval, 1.23–2.18) among adults for ischemic heart diseases (Leon et al., 1998). Although in this earlier study maternal malnutrition was the primary cause of IUGR, many other etiologies of IUGR are recognized today including placental insufficiency. Over 20 million children are born of low birth weight globally (World Health Organization, 2011), and 15% of pregnancies result in IUGR in the United States (Ananth & Vintzileos, 2009). With evidence that IUGR significantly increases the risk of adult CVD, identifying the responsible pathogenic mechanisms and timing of onset of cardiovascular disease as well as preventative and treatment strategies to reduce risks of adult CVD are critical to curtailing the major long‐term burden of CVD for affected individuals.

Placental insufficiency is a common condition in obstetrics resulting in fetal hypoxia. Fetal hypoxia has been shown to impact fetal and postnatal cardiovascular health largely through animal studies. Such studies have shown early withdrawal of cardiac myocytes from the cell cycle, apoptosis and myocyte hypertrophy (Bae, Xiao, Li, Casiano, & Zhang, 2003; Botting et al., 2018; Botting, McMillen, Forbes, Nyengaard, & Morrison, 2014; Morrison et al., 2008; Mortola, Xu, & Lauzon, 1990) and architectural changes related to energy metabolism (Gonzalez‐Tendero et al., 2013), as well as altered myocardial angiogenesis and coronary reserve (Hauton & Ousley, 2009), among other properties (Bubb et al., 2009). We have previously shown in rat models that chronic intrauterine exposure to hypoxia during the last third of pregnancy, which would be true of the human fetus in the setting of placental insufficiency, results in IUGR and leads ultimately to ventricular diastolic dysfunction and increased susceptibility to ischemic reperfusion injury by adulthood (Morton, Rueda‐Clausen, and Davidge (2011); Rueda‐Clausen, Morton, Lopaschuk, & Davidge, 2011; Xu, Williams, O'Brien, & Davidge, 2006), the latter also shown by others (Li et al., 2003). Chronic intrauterine hypoxia exposure is also associated with vascular structural and functional changes long‐term. For instance, we have demonstrated increased expression of collagen type I and III fibers, altered ß/α myosin heavy chains ratio (ß/αMHC), decreased expression of matrix metalloproteinase 2, and impaired endothelial function in mesenteric arteries of adult rats exposed to hypoxia before birth (Morton, Rueda‐Clausen, & Davidge, 2010; Rueda‐Clausen, Morton, & Davidge, 2009; Williams, Hemmings, Mitchell, McMillen, & Davidge, 2005). The timing of development of these myocardial and vascular changes and the mechanisms responsible remain poorly defined.

The aims of the current study, which utilized a previously defined rat model of prenatal hypoxia exposure (Morton et al., 2010, 2011; Rueda‐Clausen et al., 2009, 2011; Williams et al., 2005; Xu et al., 2006), were to determine if left ventricular (LV) systolic or diastolic dysfunction and increased aortic stiffness are present in early postnatal life following in utero exposure to hypoxic conditions as demonstrated in vivo using state‐of‐the‐art echo‐based techniques. We further sought to determine whether LV dysfunction correlates with histological changes in the myocardium including alterations in cardiomyocyte nuclear morphology suggestive of hypertrophy and altered collagen deposition.

## 2 MATERIALS AND METHODS

### 2.1 Animals

Female Sprague Dawley rats were obtained at 3 months of age (Charles River), acclimatized, and then mated within the animal facility. A vaginal smear obtained the following morning was examined for the presence of sperm, which we signified as day 0 of pregnancy (term ≈21 days). Throughout pregnancy, rats were housed in standard rat cages with ad libitum access to water and food (standard laboratory rat chow). On day 15 of pregnancy, rats were randomized to control (*n* = 4) or maternal hypoxia (*n* = 3) and treated as previously described in detail (Xu et al., 2006). Briefly, rats assigned to the maternal hypoxia group were placed inside a Plexiglas chamber continuously infused with nitrogen to maintain an oxygen concentration of 11.5% during the last 6 days of pregnancy. Just before birth, dams were returned to normal housing conditions (21% oxygen). At the time of birth, the litter was reduced to eight pups (four males and four females) in order to control the postnatal environment. Sixteen normoxia‐exposed and 12 hypoxia‐exposed offspring were studied. Tattoos were used to identify all pups for longitudinal study, and pups were weaned into same‐sex pairs at 3 weeks of age. Pups were weighed just prior to each echocardiographic evaluation. All dams and pups were housed in standard rat cages with 60% humidity, a 12:12 hr light:dark cycle, and ad libitum access to water and food (standard laboratory rat chow) in the animal facilities of the University of Alberta. All procedures in this study were approved by the University of Alberta Animal Welfare Committee and are in accordance with the guidelines of the Canadian Council on Animal Care and the Guide for the Care and Use of Laboratory Animals published by the US National Institutes of Health. This work aligned with the Animal Research: Reporting in Vivo Experiments (ARRIVE) Guidelines (Kilkenny, Browne, Cuthill, Emerson, & Altman, 2010).

### 2.2 Echocardiographic evaluation

Normoxia and hypoxia‐exposed pups were evaluated longitudinally by echocardiography on day 1 and day 3, week 1, week 2, week 4, and week 8 after birth. The rats were anesthetized with 1.5% isoflurane and placed in a supine position on a controlled heating pad. The chest and abdomen were shaved, and the extremities were gently fixed to the electrodes on the pad surface using tape and a highly conductive electrode gel. A single‐channel electrocardiogram signal and respiratory rate were continuously recorded on the imaging system. Body temperature was monitored by a rectal probe. Echocardiography evaluations were performed using a high‐resolution Vevo‐2100 ultrasound microscope (Visualsonics^®^) with 30–70 MHz transducers. LV dimensions, LV systolic and diastolic function, and aortic stiffness were assessed using 2‐dimensional and M‐mode imaging and pulsed and tissue Doppler‐based modalities.

LV dimensions and function were analyzed using protocol‐based measurements and calculations as previously described for rodents (Watson, Sheth, Denyer, & Dostal, 2004). For each evaluation, dimensions of the LV cavity diameter and the thickness of its walls were assessed using a parasternal short‐axis view of the heart with the M‐mode beam positioned just below the mitral leaflet tips, perpendicular to the long axis of the ventricle and centered in the short axis. LV dimensions were corrected for the weight of the pup. LV systolic function was evaluated by estimating ejection fraction, shortening fraction and stroke volume/weight from images obtained in M‐mode of the LV short axis, rate‐corrected velocity of circumferential fiber shortening (VCFc) (Fortuin, Hood, & Craige, 1972), and tissue Doppler S’ at the septum and LV lateral wall. Vcfc measures the velocity of dimensional changes during ejection which is calculated by dividing the percentage fractional shortening by the ejection time corrected for heart rate variability (Fortuin et al., 1972). Ventricular diastolic function was assessed by describing the transmitral pulse wave Doppler signal E and A wave velocities and E/A ratio. Tissue Doppler measures of diastolic function were also assessed at the septum and LV lateral wall and included E’, A’, E’/A’, ratio, and isovolumic relaxation time (IVRT).

Aortic stiffness was determined through the calculation of the pulse wave velocity (PWV) as previously described in humans (Sandor et al., 2003) and rats (Morgan, Casabianca, Khouri, & Kalinoski, 2014). At the time of echo assessment, ascending aortic pulse wave Doppler tracings were obtained using immediately sequentially acquired samples in the ascending aorta (T1) just above the aortic valve and the descending abdominal aorta just at the diaphragm (T2) and just above the bifurcation into iliac arteries (T2). All measures were averaged over three cardiac cycles. We calculated transit time (T2‐T1), measured from the onset of the R wave in the QRS waveform to the onset of flow, and together with the distance from T1 to T2 along the aortic arch, we calculated the PWV using the equation: PWV = distance/ (T2‐T1).

### 2.3 Histology, morphometry, and quantitative analysis

The heart was fixed in toto*,* and cross sections of the ventricles were taken at a standard distance from the base and apex of the heart specimens. The myocardium from week 2 and week 4 littermates of the noninvasively evaluated hypoxia‐exposed and control pups was fixed in 10% neutral buffer formalin, embedded in paraffin and sectioned. Hematoxylin and eosin (H&E) staining was performed to evaluate nuclear size and shape for assessment of cardiomyocyte hypertrophy (Chiu & Sergi, 2014) and Picro‐sirius red staining was used to assess for fibrosis (Farris et al., 2011; Hadi et al., 2011; Huang et al., 2013; Street et al., 2014), specifically identifying the collagen fibers and their density. Picro‐sirius red staining is considered ideal for collagen staining because it does not fade, is selective and highly reproducible, more so than Masson's trichrome or collagen immunohistochemistry (Farris et al., 2011). All histological assessments were performed by a single investigator (CS) who was blinded to pregnancy and intrauterine exposure. As manual measurement and counting of fibers can be error‐prone, cardiomyocyte morphology and collagen I/III deposition and arrangement were assessed using an operator‐interactive, semi‐automated method for quantification of fiber data as previously reported (Amella et al., 2008; Street et al., 2014). We chose to use this method to assess cardiomyocyte characteristics (nuclear number, size, area and shape and collagen fibers) as past studies have suggested this method is equivalent to Cavalieri stereology with minimal differences (Marcos, Braganca, & Fontes‐Sousa, 2015). The parameters measured from H & E stained sections were the variation of area, perimeter, and width of the nuclei as well as the variation of their angle, circularity, and Feret, skewness and kurtosis. The Feret diameter is the longest distance between any two points along the selection boundary. Collagen fiber number and both perimeter and thickness were assessed.

### 2.4 Statistical analyses

Means and standard errors were calculated for the 2D and Doppler parameters of LV function and aortic PWV at the different developmental ages. Comparison between the normoxia and hypoxia‐exposed groups at each age was performed with two‐sided Student's *t* test (with Bonferroni's correction for the significance level; *p*‐values < .008 were considered as statistically significant). Longitudinal changes of parameters were estimated by using a mixed‐ANOVA (analysis of variance) design with repeated measures. The effect of litter was analyzed using mixed‐effects models considering the pups to be clustered into litters to determine if the outcomes from the mixed repeated ANOVA, with pups being considered independent of litter, were different. Comparisons between week 2 and week 4 hypoxia and control pups for nuclear characteristics and collagen fiber counts and dimensions were assessed by 2‐way ANOVA and Tukey post hoc test. We used licensed copies of SPSS v24 and GraphPad Prism for performing the statistical analysis.

## 3 RESULTS

### 3.1 Developmental changes in LV dimensions and function in the normal rat

In control rat pups, LV lateral (posterior) wall and septal (not shown) wall thickness in diastole corrected for weight were constant between day 1 and day 3 and subsequently decreased. LV end‐diastolic diameter corrected for weight decreased with age. Heart rate increased progressively with age to week 4. LV stroke volume corrected for weight decreased with age. With respect to systolic function, ejection fraction remained constant from day 1 to week 8, whereas rate‐corrected velocity of circumferential fiber shortening (VCFc) and systolic myocardial velocities (S’) of the lateral wall and septum progressively increased with age (Table 1). LV diastolic function parameters demonstrated a progressive increase in ventricular inflow E and A wave pulsed Doppler and E’ and A’ annular tissue velocities with age with greater increases in early diastolic (E and E’) compared to late diastolic (A and A’, reflecting atrial contraction) velocities, particularly during the period from day 1 to week 4 (Table 2). Although the isovolumic relaxation (IVRT), another measure of diastolic function, decreased with age in the normal rat, its duration relative to the cardiac cycle length (R‐R) did not change significantly throughout the study period.

**Table 1:** General and LV Systolic function parameters from day 1 to week 8 after birth

Parameter	Day 1	Day 3	Week 1	Week 2	Week 4	Week 8	Overall time effect
Weight (g)							
Control	9.2 ± 0.4	10.9 ± 0.3	17.8 ± 0.8	37.4 ± 0.7	108.6 ± 2.5	321.6 ± 18.9	
Hypoxia	7.6 ± 0.4* ^,^ a	10.0 ± 0.3	16.2 ± 0.8	33.1 ± 0.7	104.0 ± 2.5	311.9 ± 18.9	*p* = .000
LV EDD/wt (mm/kg)							
Control	0.31 ± 0.01	0.26 ± 0.01	0.19 ± 0.00	0.12 ± 0.00	0.05 ± 0.00	0.024 ± 0.001	
Hypoxia	0.333 ± 0.02	0.29 ± 0.01	0.20 ± 0.01	0.12 ± 0.00	0.06 ± 00	0.024 ± 0.001	*p* = .000
LV PWd/wt (mm/kg)							
Control	0.06 ± 0.01	0.06 ± 0.00	0.05 ± 0.00	0.02 ± 0.00	0.01 ± 0.00	0.005 ± 0.000	
Hypoxia	0.06 ± 0.00	0.07 ± 0.00	0.04 ± 0.00	0.03 ± 0.00	0.01 ± 0.00	0.005 ± 0.000	*p* = .000
Heart rate (bpm)							
Control	304 ± 6	349 ± 8	362 ± 12	382 ± 7	435 ± 9	403 ± 8	
Hypoxia	260 ± 9*	342 ± 4	352 ± 5	400 ± 9	410 ± 13	406 ± 7	*p* = .000
Stroke volume/wt (µl/kg)							
Control	2.61 ± 0.13	2.46 ± 0.11	2.17 ± 0.10	1.76 ± 0.09	1.22 ± 0.07	0.83 ± 0.04	
Hypoxia	2.78 ± 0.14	2.78 ± 0.14	2.12 ± 0.12	1.69 ± 0.10	1.28 ± 0.08	0.82 ± 0.05	*p* = .000
VCFc (circ/s)							
Control	2.12 ± 0.1	2.47 ± 0.1	2.57 ± 0.07	2.36 ± 0.1	3.00 ± 0.18	3.58 ± 0.2	
Hypoxia	1.84 ± 0.14	2.62 ± 0.15	2.80 ± 0.19	3.05 ± 0.15*	2.76 ± 0.17	3.34 ± 0.14	*p* = .000
Septal S’ vel (mm/s)							
Control	14.5 ± 1.0	17.8 ± 0.8	20.3 ± 0.8	18.9 ± 0.8	36.6 ± 1.7	52.3 ± 1.8	
Hypoxia	14.1 ± 0.6	17.4 ± 0.6	19.4 ± 0.8	23.6 ± 0.7*	36.9 ± 1.7	55.9 ± 3.1	*p* = .000
LV LW S’ vel (mm/s)							
Control	15.3 ± 0.5	18.5 ± 0.5	20.8 ± 0.5	18.9 ± 0.7	32.6 ± 1.3	47.6 ± 1.7	
Hypoxia	15.1 ± 0.8	18.6 ± 0.4	20.2 ± 0.7	23.5 ± 0.6*	37.8 ± 2.3	56.7 ± 2.6*	*p* = .000
AOd‐PWV (m/s)							
Control	2.9 ± 0.2	2.4 ± 0.1	2.1 ± 0.1	2.2 ± 0.1	3.6 ± 0.2	3.9 ± 0.2	
Hypoxia	12.2 ± 0.3.1*	9.6 ± 1.4*	8.6 ± 2.5*	3.6 ± 0.3*	4.5 ± 0.5	5.4 ± 0.3*	*p* = .000

Note

LV‐left ventricular, PWd‐posterior wall thickness in diastole, IVSd‐interventricular septal wall thickness in diastole, LW‐left ventricular lateral wall, VCFc‐rate‐corrected velocity of circumferential fiber shortening, vel‐velocity. All data are presented as mean ± *SE*. Highlighted variables represent those with differences between prenatal hypoxia‐exposed and control pups at specific ages.

a No statistical difference when the effect of litter was incorporated into the analysis.

* *p* ≤ .008 for differences between hypoxia exposed and control pups at a given age.

**Table 2:** Changes in LV diastolic function and aortic stiffness assessed by mitral valve pulse flow and tissue Doppler

Parameter	Day 1	Day 3	Week 1	Week 2	Week 4	Week 8	Overall time effect
MV E vel ( mm/s)							
Control	336 ± 15	449 ± 21	542 ± 19	658 ± 20	920 ± 39	1,035 ± 30	
Hypoxia	314 ± 22	524 ± 16	541 ± 24	703 ± 26	847 ± 38	868 ± 32*	*p* = .000
MV A vel (mm/s)							
Control	436 ± 17	493 ± 30	475 ± 21	459 ± 17	575 ± 20	646 ± 30	
Hypoxia	408 ± 22	596 ± 20	545 ± 20	528 ± 25	619 ± 25	650 ± 35	*p* = .000
LV LW E’ vel (mm/s)							
Control	13.3 ± 1.6	13.6 ± 1.2	16.4 ± 0.6	20.3 ± 1.0	44.3 ± 2.4	52.9 ± 2.8	
Hypoxia	9.5 ± 0.7	12.3 ± 0.6	14.4 ± 0.5	26.0 ± 1.7* ^,^ a	36.6 ± 2.9	52.6 ± 3.5	*p* = .000
LV LW A’ vel (mm/s)							
Control	18.8 ± 1.9	18.4 ± 1.4	19.9 ± 0.8	19.4 ± 1.3	39.2 ± 2.0	46.4 ± 2.5	
Hypoxia	16.7 ± 1.0	18.9 ± 0.7	21.2 ± 0.5	29.8 ± 0.8*	43.4 ± 3.4	65.3 ± 5.0*	*p* = .000
LV LW E’/A’ vel							
Control	0.70 ± 0.02	0.73 ± 0.02	0.83 ± 0.03	1.08 ± 0.06	1.14 ± 0.04	1.16 ± 0.05	
Hypoxia	0.58 ± 0.03	0.65 ± 0.03	0.68 ± 0.02*	0.89 ± 0.08	0.85 ± 0.02*	0.82 ± 0.03*	*p* = .000
AOd‐PWV (m/s)							
Control	2.9 ± 0.2	2.4 ± 0.1	2.1 ± 0.1	2.2 ± 0.1	3.6 ± 0.2	3.9 ± 0.2	
Hypoxia	12.2 ± 0.3.1*	9.6 ± 1.4*	8.6 ± 2.5*	3.6 ± 0.3*	4.5 ± 0.5	5.4 ± 0.3*	*p* = .000

Note

AOd‐pulse wave velocity (PWV) at the diaphragm; MV‐mitral valve; LV LW‐left ventricle lateral wall, vel‐peak velocity. All data are presented as mean ± *SE*. Highlighted variables represent those with differences between prenatal hypoxia‐exposed and control pups at specific ages.

a No statistical difference when the effect of litter was incorporated into the analysis.

* *p* < .008 for differences between hypoxia exposed and control pups at a given age.

### 3.2 Impact of fetal hypoxia on early LV function

**Figure 1:**
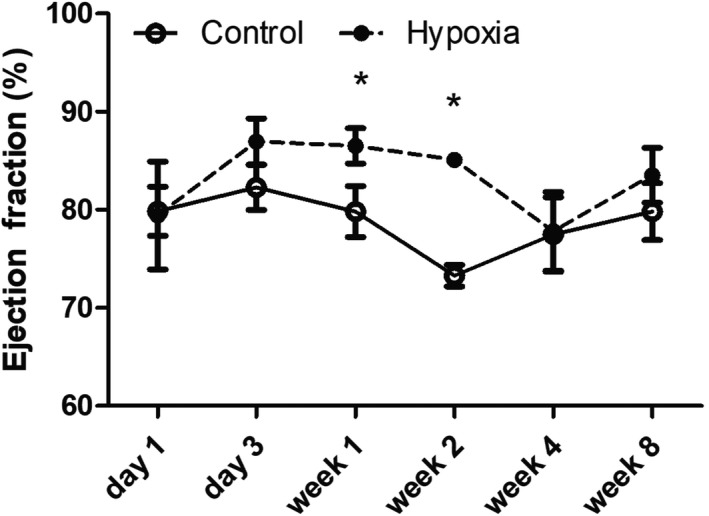
Evolution of LV Ejection Fraction in Hypoxia versus Normoxia‐Exposed Pups. Transiently increased LV ejection fraction was observed in prenatal hypoxia‐exposed rat pups at weeks 1 and 2. All data are presented as mean ± *SE* (*p < .008)

LV lateral and septal (not shown) wall thickness in diastole and LV end‐diastolic diameter corrected for weight did not differ between prenatal hypoxia‐exposed and control pups. LV stroke volume corrected for weight also did not differ. Heart rates were significantly lower in the fetal hypoxia rats at day 1 compared to controls but did not differ at any other stage. LV systolic function parameters (Table 1 and Figure 1) were increased by week 1 (LV ejection fraction, Figure 1) and week 2 (all variables (VCFc, septal and lateral wall S’ velocities) in hypoxia relative to the control group (Table 1). LV systolic lateral wall velocities remained significantly increased in hypoxia‐exposed pups at week 8.

**Figure 2:**
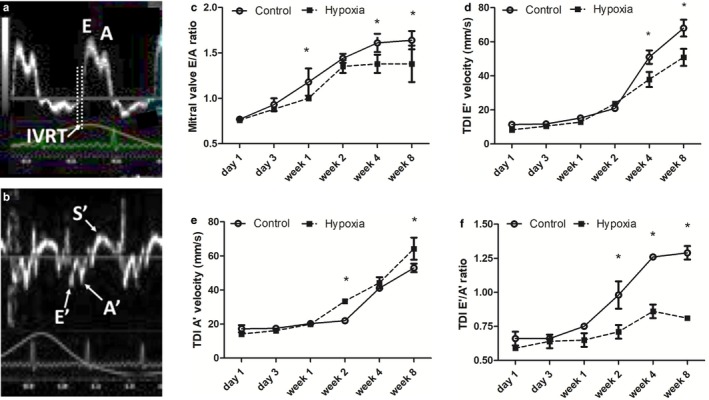
Evolution of LV Inflow and tissue Doppler velocities in Prenatal Hypoxia versus Normoxia Pups. (a) and (b) demonstrate representative mitral valve inflow (upper) and tissue velocity (lower) Doppler tracings acquired in the rat pups. Graphs (c) through (f) demonstrate the evolution of the mitral valve E/A wave ratio and septal diastolic velocities in hypoxia and normoxia‐exposed pups. (c) Although the LV inflow E/A wave increased in both intrauterine hypoxia and normoxia‐exposed pups with age, this increase was significantly less in the former due to less of an increase in early filling velocities and a greater reliance on filling with atrial systole. (e) Early diastolic septal E’ velocity increased in both hypoxia‐exposed and control pups with age, but was significantly lower in hypoxia‐exposed pups by week 4 and week 8. (e) Late diastolic septal (A’) velocities also increased in both hypoxia‐exposed and control pups from day 1 to week 8 with statistically higher velocities at week 2 and week 8 in the former. (f) Septal E’/A’ wave ratios increased in both groups, but, as a consequence of less change in E’ and higher A’ velocities, were progressively lower in hypoxia‐exposed pups from week 2 onward. All data are presented as mean ± *SE* (*p < .008)

**Figure 3:**
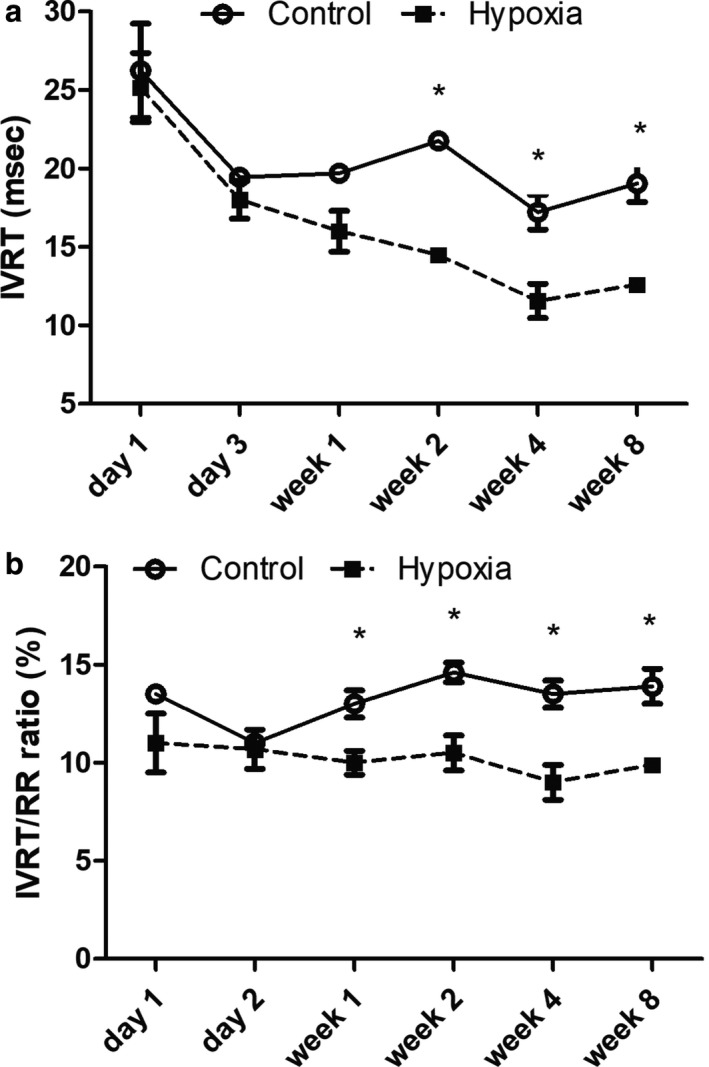
Evolution of LV Isovolumic Relaxation Time in Prenatal Hypoxia versus Normoxia Pups. Isovolumic relaxation time (IVRT) became progressively shorter in prenatal hypoxia‐exposed pups from week 1 compared to controls. (a) IVRT duration decreased in both hypoxia‐exposed and control pups from day 1 to week 8, but was significantly lower in hypoxia‐exposed pups from week 2 onward. (b) The IVRT duration relative to the R‐R interval remained constant in hypoxia‐exposed and control pups but was significantly lower in hypoxia‐exposed pups from week 1 onward. IVRT‐isovolumic relaxation time and R‐R‐cardiac cycle length (*p = .008)

Rat pups exposed to a hypoxic intrauterine environment demonstrated abnormalities of diastolic function based on pulsed and tissue Doppler profiles and velocities that were progressive (Table 2, Figure 2). These abnormalities included evolution toward greater late ventricular filling (A) and tissue velocities during atrial systole (A’), and a shortened period of isovolumic relaxation. Prenatal hypoxia‐exposed pups demonstrated normal early (E) LV peak filling velocities until week 8, at which time they were mildly decreased. Late diastolic LV inflow velocities occurring with atrial systole (A) tended to be increased in hypoxia rats resulting in a significant reduction in the E/A wave ratio by weeks 1, 4, and 8, suggesting greater filling during atrial contraction compared to controls (Figure 2a‐c). Tissue Doppler demonstrated a decrease in early diastolic septal velocities (E’), and an increase in septal (Figure 2d,e) and lateral wall velocities (Table 2) in atrial systole (A’) in the hypoxia group. As a consequence of the latter changes, a significant and progressive decrease in E’/A’ ratio for septal and lateral walls from week 2 onward was observed (Figure 2f). IVRT was significantly shortened in hypoxia rat pups from week 2 onward, and the IVRT duration relative to the R‐R interval was significantly shorter from week 1 in hypoxia rats (Figure 3a,b). By incorporating the hierarchical (clustered) structure of the data to account for the impact of litter, only two variables differed from the analysis when the LV dimensions and function for all pups were considered independently. Pup weight on day 1 was no longer statistically significant, and differences between hypoxia‐exposed and control pups for LV lateral wall E’ at week 2 no longer reached statistical significance. Analyses for all other variables remained unchanged.

### 3.3 Impact of fetal hypoxia on aortic stiffness

**Figure 4:**
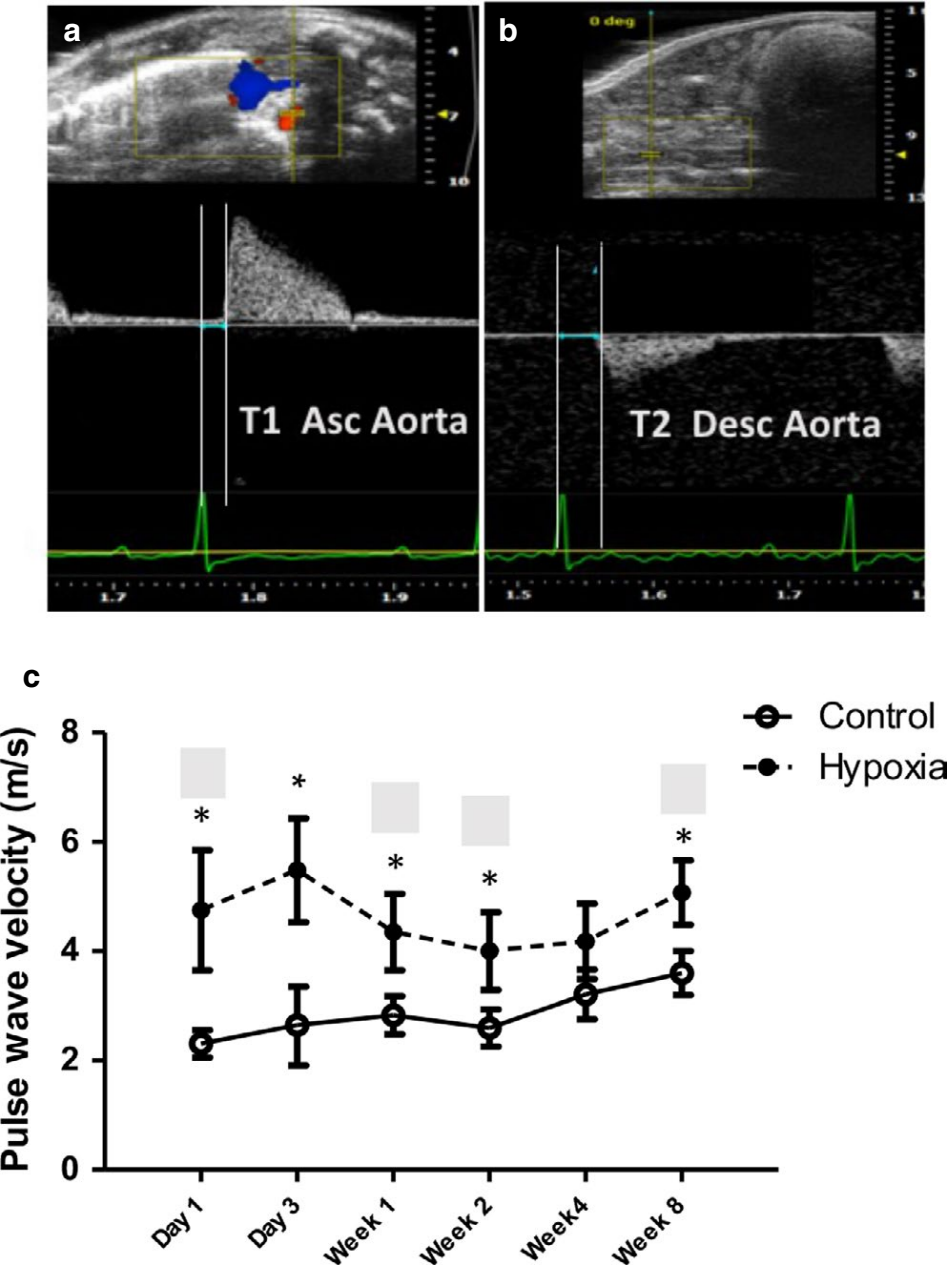
Evolution of Aortic Pulse Wave Velocity in Prenatal Hypoxia versus Normoxia Pups. (a) and (b) demonstrate the technique of measuring T1 and T2 for the aortic pulse wave velocity at the ascending aorta and aortic bifurcation, respectively, as a measure of aortic stiffness. Both time intervals are measured from the onset of the QRS to the onset of flow. The aortic pulse wave velocity (PWV) was then calculated by the distance between the two points of measure divided by T2‐T1 in M/s. (c) The aortic PWV with T2 measured just above the aortic bifurcation was most increased at day 1 and day 3 in prenatal hypoxia‐exposed rat pups but was still increased at week 8 compared to controls (*<.008)

Control rat pups demonstrated a slight increase in aortic pulse wave velocity (PWV) with age after week 2. In contrast, intrauterine hypoxia‐exposed pups demonstrated significantly increased aortic PWV at the earliest developmental stages, approaching controls by week 2, and remaining stable but higher thereafter (Figure 4), suggesting increased aortic stiffness.

### 3.4 Impact of fetal hypoxia on the early LV myocardium

**Figure 5:**
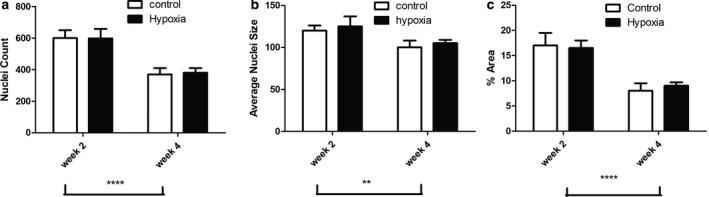
Myocyte Nuclei Count, Size & Area of the Myocyte in Prenatal Hypoxia and Normoxia Pups. Comparison of a) nuclear count, b) nuclear size, and c) proportion of the nuclear area relative to the myocyte area at 2 and 4 weeks in hypoxia‐exposed and control rat pups. There was a significant decrease in nuclear count and size with age but no significant difference between hypoxia and normoxia‐exposed pups. Data were analyzed by 2‐way ANOVA: ** *p* < .01, ****p < .0001 group effect of offspring age

**Figure 6:**
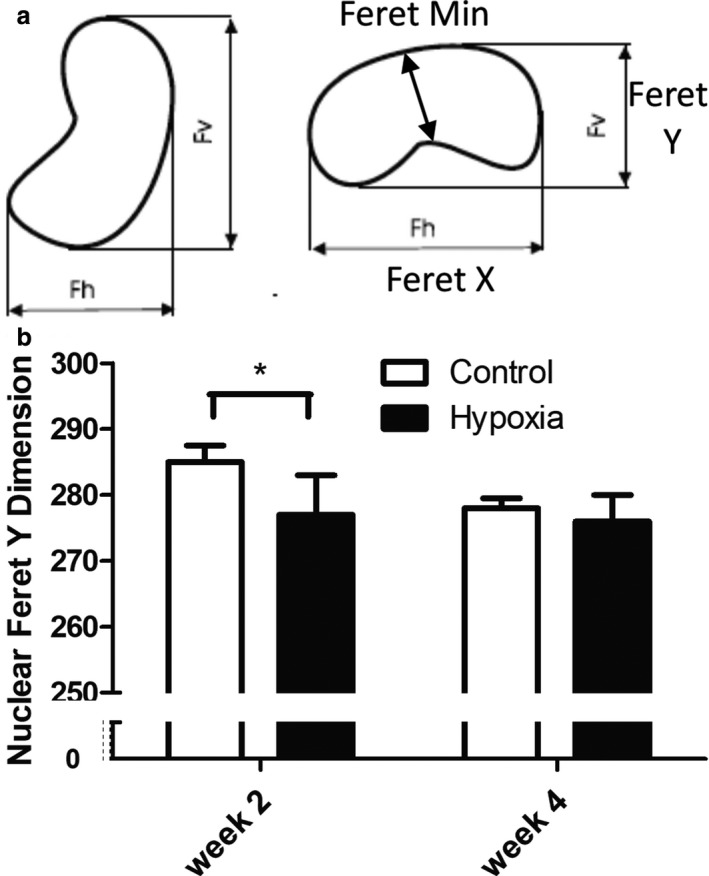
Myocyte Nuclear Shape in Prenatal Hypoxia and Normoxia Pups. Comparison of myocyte nuclear shape between hypoxia‐exposed and control rat pups at weeks 2 and 4: a) Diagram demonstrating the nuclear dimensions assessed. b) Only differences in Feret Y dimension were observed between the groups with a reduced dimension in hypoxia‐exposed pups at week 2 (*p = .003 by *t* test)

With age in both hypoxia‐exposed and control rat pups, there was a decrease in nuclei counts, total nuclei area, and the percentage of nuclear area relative to the cardiomyocyte area (Figure 5). Although these changes in the myocardium did not differ between the groups, the shape of the nucleus differed with a decrease in ventricular nuclei Feret Y dimension at week 2 in hypoxia‐exposed pups which normalized by week 4 (Figure 6). All other measures of nuclear morphology did not differ with age or between hypoxia and control pups.

**Figure 7:**
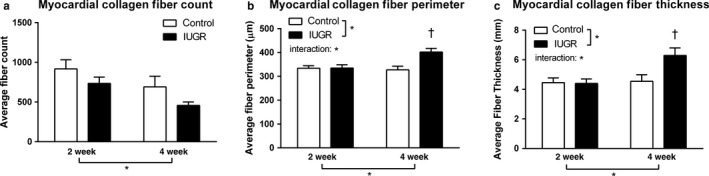
LV Myocardial Collagen in Prenatal Hypoxia and Normoxia Pups. Comparison of collagen fiber deposition between hypoxia‐exposed and control rat pups at week 2 and week 4: Collagen fiber (a) count, (b) perimeter, and (c) thickness at week 2 and week 4 in hypoxia‐exposed and control rat pups are demonstrated. Although both groups demonstrated a decrease in fiber number with age, there was a significant increase in fiber perimeter and thickness by week 4 in rat pups exposed to hypoxia before birth compared to normoxic controls which showed no significant difference in fiber size between week 2 and week 4. Data were analyzed by 2‐way ANOVA: *p < .05 group effect of prenatal environment, offspring age or interaction of the factors. Tukey's post hoc test: †*p* < .05 versus all other groups

With respect to collagen fibers, there was a decrease in the total number of fibers in both groups with age, the extent of which did not differ between hypoxia‐exposed and control pups; however, collagen fiber perimeter and thickness increased significantly more so with age in the hypoxia‐exposed pups (Figure 7). Changes in the quality of collagen, including increased fiber thickness, are recognized pathological features associated with abnormalities of diastolic function in aging and pathological states (Bradshaw et al., 2009; Brower et al., 2006; Lopez, Querejeta, Gonzalez, & Larman, 2012; Yarbrough et al., 2012).

## 4 DISCUSSION

Our longitudinal study is the first to document in vivo the evolution of myocardial function in the healthy developing rat and the impact of exposure to a hypoxic intrauterine environment on myocardial function and aortic stiffness during early postnatal stages. Rodent models are increasingly used to study diseases of infancy and childhood. The availability of specialized ultrasound biomicroscopes with high frequency, high‐resolution transducers that permit in vivo assessments using state‐of‐the‐art echocardiography‐based modalities has made it now possible to investigate developmental changes in myocardial function longitudinally from very early stages in a way that is comparable to techniques used clinically in humans. Understanding the evolution of myocardial function and differences in function compared to that of humans provides insight into the translational capabilities of rodent models for pediatric and adult cardiovascular disease (Chien, 1996).

Only one previous report has documented postnatal developmental changes in myocardial function in murine models using echocardiographic techniques, and this particular study only focused on LV inflow Doppler in mice from day 1 to week 12 (Zhou, Foster, Parkes, & Adamson, 2003). Our study examined both systolic and diastolic LV functional changes in early development using standard clinical parameters. Systolic function was assessed using ejection fraction, Vcfc, and systolic tissue Doppler velocities (S’). Although in the human (Alp et al., 2012; Henry et al., 1978) and the rat, LV ejection fraction does not significantly change through early development, there are differences between humans and rats in Vcfc. In humans, Vcfc has been shown to be highest in the newborn, decreasing over the first 2 years and then plateauing thereafter through to adulthood (Colan, Borow, & Neumann, 1984; Colan, Parness, Spevak, & Sanders, 1992), whereas in the rat we observed a steady increase with age through week 8, the equivalence of early adolescence. Vcfc is sensitive to contractility and is preload independent (Colan et al., 1984). Our observations that suggest early stages are associated with increasing contractility in the rat, also documented in the rabbit (Nakanishi & Jarmakani, 1984) and lamb (Teitel et al., 1985), may relate to species‐specific differences in myocardial composition, the efficiency of and transition in energy metabolism, as well as changes in sympathetic and parasympathetic function (Carnevali & Sgoifo, 2014). Although contractility itself has not been examined previously in vivo in the developing rat, structural and biochemical changes occur during this period that potentially contribute to augmented contractility including increased total creatine kinase activity and binding of creatine kinase enzymes to myofibrils and mitochondria, increased lactate dehydrogenase (H subunit) activities (CarnevLetout et al., 2005), and changes in the ventricular content of α and ß isoforms of myosin heavy chain (MHC) (Dechesne, Leger, & Leger, 1987; Lompre et al., 1981). Finally, phospholipids and fatty acid content of the myocardium increase especially in the first 3 weeks after birth in the rat, which may augment cell signaling (Novák, Tvrzická, Hamplová, Kolár, & Nováková, 2006). S’ velocities, which increased in the early developmental stages of the rat, also increase progressively with age in humans (Roberson, Cui, Chen, Madronero, & Cuneo, 2007). Although changes in S’ could reflect changes in intrinsic myocardial contractility (Eidem et al., 2004; Gorcsan, Strum, Mandarino, Gulati, & Pinsky, 1997), increasing preload that would accompany growth likely also plays an important role. Interestingly, in infants and children S’ velocities negatively correlate with heart rate (Schmitz, Koch, Bein, & Brockmeier, 1998), whereas we observed a progressive increase in S’ velocities despite increasing heart rates, which may again highlight developmental differences in the different species.

With respect to loading and diastolic properties, peak mitral valve E and A wave Doppler velocities, and septal and lateral wall E’ and A’ velocities progressively increased with age in the rat with a greater increase in early relative to late diastolic velocities, resulting in increased E/A and E’/A’ ratios through week 4. In humans, early peak filling (E) and annular (E’) velocities acutely increase through the first year of life and continue to increase at a slower rate through adolescence (Eidem et al., 2004; Schmitz et al., 1998). LV inflow velocities during atrial systole, however, decrease throughout childhood and atrial systolic septal and lateral wall tissue velocities do not change with age and are unrelated to end‐diastolic dimensions (Eidem et al., 2004). Developmental differences in myocardial maturation and heart rate likely contribute directly and indirectly to the different patterns of evolution of these parameters in rats and humans. In humans, for instance, decreasing heart rate may provide more time for early filling resulting in progressively increasing E and E’ velocities (Eidem et al., 2004). Finally, IVRT (both absolute and relative to the cardiac cycle length) progressively decreases in the developing rat, a finding suggestive of more efficient relaxation with age (Cappelli, Tortelli, Zani, Poggesi, & Reggiani, 1988), whereas in humans, the absolute IVRT remains unchanged from neonatal to adolescent stages, shortening only relative to the cardiac cycle length (Eidem et al., 2004).

### 4.1 Fetal hypoxia and early postnatal myocardial function & aortic stiffness

Chronic intrauterine exposure to hypoxia led to several changes in cardiac function in our rat model, some transient (heart rate and systolic function) and others more progressive (LV diastolic dysfunction). We observed a brief period of bradycardia in the hypoxia‐exposed pups only on day 1 which normalized thereafter. The observed bradycardia could relate to altered fetal parasympathetic activity (Portbury et al., 2003), which we suspect could carry over to the immediate newborn period, resolving subsequently with exposure to normoxic conditions. Following intrauterine hypoxia exposure, rat pups demonstrated no significant change in LV wall thickness. They did demonstrate increased systolic performance after day 1 which reached statistical significance only by week 1 (ejection fraction) and 2 (all parameters) and subsequently normalized with the exception of LV lateral wall velocities. With the exception of Vcfc, ejection fraction and S’ are load‐dependent variables. While we had not observed an increase in end‐diastolic dimensions as a surrogate for LV preload, changes in afterload may have contributed to transient enhancement of systolic function. Both aortic stiffness and vasoconstriction contribute to LV afterload. In prenatal hypoxia‐exposed pups, we found aortic stiffness to be greatest immediately after birth, decreasing by week 1 but remaining greater than in controls thereafter. Fetal hypoxia has also been shown to be associated with vasoconstriction, at least in part due to sympathetic stimulation (Rouwet et al., 2002), which may resolve with normoxic conditions after birth. Perhaps with aortic/arterial remodeling leading to decreased stiffness by week 1 (albeit still abnormal) and decreased vasoconstriction, there was a consequent temporary increase in systolic function. Despite an increase in systolic function, we were unable to demonstrate a difference in stroke volume/weight which may relate to the effect of persistent, albeit less afterload and no augmented preload. Additional contributing factors could include increased sensitivity of ß adrenergic receptors observed transiently postnatally following chronic fetal hypoxia exposure (Lindgren & Altimiras, 2009) which could have contributed to increased VCfc as well. However, one would expect this effect would be more apparent acutely following delivery when circulating catecholamines are highest (Habib et al., 1991), and that this would be associated with augmented stroke volume and increased heart rate which were not observed. The long‐term relevance of these changes remains unclear, particularly given normalization of clinical parameters by week 8.

Previous in vitro investigations have documented abnormalities of diastolic function in 11‐week‐old rats exposed to a hypoxic intrauterine environment before birth (Hauton & Ousley, 2009). Our in vivo observations suggest that diastolic dysfunction begins early in life and is progressive. We found early LV filling velocities (E) to be significantly lower than controls by week 8 and a progressive decrease in early relative to late LV filling velocities (E/A) from week 1 onward. Myocardial wall velocities demonstrated even greater differences in early versus late diastolic motion with a progressive decrease in E’/A’ compared to controls from week 4 onward. These findings are most consistent with abnormal (prolonged) relaxation, with increased dependence of LV filling on atrial contraction (Cohen, Pietrolungo, & Thomas, 1996; Garcia, Thomas, & Klein, 1998; Gibson & Francis, 2003; Oh, Park, & Nagueh, 2011); however, LV relaxation abnormalities that are more severe in human disease are usually accompanied by prolonged IVRT (Cohen et al., 1996; Garcia et al., 1998; Gibson & Francis, 2003; Oh et al., 2011). In contrast, we found intrauterine hypoxia exposure to be associated with reduced IVRT relative to the cardiac cycle length as early as week 1, a finding observed in restrictive myocardial disease (Cohen et al., 1996), becoming progressively different from controls thereafter. Shortened IVRT has been observed in adult rats exposed to hypoxia before birth (Rueda‐Clausen et al., 2011) suggesting that the onset of this pathology could occur quite early in development. A concomitant decrease in E and E’ and increase in A and A’ with shortened IVRT could reflect a combination of abnormalities of myocardial relaxation and compliance, the latter potentially resulting in greater restoring forces with rapid untwisting in early diastole. Echo‐based speckle tracking techniques available on state‐of‐the‐art ultrasound biomicroscopes coupled with an exploration of histological and biochemical changes in myocardial components can be used to further explore the relative contributions of altered relaxation and compliance in myocardial mechanics that may play an important role in myocardial health long‐term.

Extracellular matrix remodeling within the myocardium has been identified previously following intrauterine hypoxia exposure which may be a key contributor to diastolic dysfunction. Increased type 1 collagen, greater total collagen content, and increased cross‐linking of collagen have been demonstrated in day 7 rat pups with hypoxia exposure before birth (Tong, Xue, Li, & Zhang, 2011). Abnormalities in the quality and quantity of collagen are known to contribute importantly to myocardial stiffness in various disease states including aging (Bradshaw et al., 2009; Brower et al., 2006; Lopez et al., 2012; Yarbrough et al., 2012). Although the quantity was not increased grossly, we found thicker collagen fibers in pups with hypoxia exposure which may contribute to greater myocardial stiffness. Altered expression patterns of matrix metalloproteinase and tissue inhibitor of metalloproteinase (Tong et al., 2011) could negatively impact collagen remodeling and contribute to the progressive diastolic pathology we observed. Previous investigations suggest that changes in myocardial extracellular matrix and consequent function persist and may even be progressive later in life (Xu et al., 2006). By 12 months, the equivalent of a middle‐aged human, rats exposed to a hypoxic prenatal environment ultimately reveal Doppler‐based findings that are consistent with LV restrictive physiology including rapid offset of early diastolic filling (short deceleration time) and reduced filling in atrial systole (Li et al., 2003).

Fetal hypoxia has been shown to contribute to reduced cardiomyocyte proliferation and increased apoptosis, cardiomyocyte hypertrophy, and ventricular wall thinning (Bae et al., 2003; Sugishita, Watanabe, & Fisher, 2004). In the present study, we did not find obvious LV hypertrophy based on in vivo measures of LV diastolic wall thickness and percent nuclear area (size) to myocyte area (size). We had also not found no evidence for an increase in cardiomyocyte number (hyperplasia) in hypoxia‐exposed rat pups relative to controls by weeks 2 and 4, a finding demonstrated previously in late gestation fetal rats exposed to hypoxia (Corstius et al., 2005). That rat cardiomyocytes continue to proliferate until day 4 (Li, Wang, Capasso, & Gerdes, 1996), there may be potential for postnatal recovery after prenatal hypoxia exposure once delivered in normoxic conditions as previously suggested (Botting et al., 2012). We did observe a change in nuclear shape at week 2 which normalized by week 4 that could relate indirectly to changes in extracellular matrix and altered gene expression (Gerdes, Liu, & Zimmer, 1994; Kim, Li, Phillip, Wirtz, & Sun, 2015). How this change in nuclear shape contributes to the structural and functional changes at an organ, histological, cellular, and molecular level requires further study. We chose to evaluate nuclear size and morphology as cardiomyocyte borders are often overlapping and sections are not planar. The blue staining of the nucleus on H & E is easy to identify and skeletonize (Rueden et al., 2017; Schneider, Rasband, & Eliceiri, 2012). In human cardiac pathology, hypertrophic cardiomyocytes show an increase in the nuclear size, which we did not observe, but they may also take on altered nuclear shapes believed to represent changes in biosynthetic activity (Nozynski et al., 2007).

Some of the changes in myocardial function could be secondary to altered ventricular afterload related to abnormal vascular health. Chronic fetal hypoxia in animal studies has been shown to contribute to increased peripheral arterial wall thickness and to alter the matrix of arterial walls with increased collagen and decreased elastin content before birth (Nozynski et al., 2007; Thompson, Richardson, Gagnon, & Regnault, 2011). Other models of IUGR, including uterine artery ligation, have also induced increased peripheral arterial stiffness in rats even by week 3 due to vascular remodeling (Dodson et al., 2017). We have now confirmed the presence of increased aortic stiffness at the earliest postnatal stages in vivo, worse within the first few days after birth but persistently abnormal by week 8. Chronic increases in arterial stiffness, which increase end‐systolic LV wall stress, have been shown in rat models to induce LV hypertrophy and lead to increased myocardial collagen content and fibrosis (Lartaud‐Idjouadiene, Lompre´, Kieffer, Colas, & Atkinson, 1999) which could contribute to the observed abnormalities of diastolic function and the histological changes observed both early and long term.

### 4.2 Clinical relevance

The developmental stages we examined in the rat correspond in humans to approximately the late fetal (days 1 and 3), the neonatal period (week 1), early infancy (week 2), early childhood (week 4), and early adolescence (week 8) in humans (Sengupta, 2011). Translating our work to humans exposed to hypoxic intrauterine conditions before birth, such as placental insufficiency with growth restriction, one might expect diastolic dysfunction to be present beyond the newborn period, but still in early infancy. Several studies have been performed in neonates (Sehgal, Doctor, & Menahem, 2013), infants (Altın et al., 2012), and children (Crispi et al., 2010) with a history of IUGR. Some have reported contradictory findings and this could relate to confounding clinical pathologies that concomitantly impact fetal and neonatal myocardial function, including preterm birth and other comorbidities. Echocardiograms performed during the first week after birth among similarly affected neonates have shown increased LV E/A ratio (Altın et al., 2012) and E’/A’ ratio (Altın et al., 2012) with decreased A velocities (Altın et al., 2012; Sehgal et al., 2013). Longitudinal analysis of formerly growth‐restricted fetuses has shown normalization of E/A and E’/A’ ratio by 3 months (Altın et al., 2012). A single prospective cohort study performed at a mean age of 5 years showed normalized E/A ratio and A velocity but lowered E’ velocity ratio in children who had been severely growth‐restricted before birth due to placental insufficiency (Crispi et al., 2010). Although the latter finding could be more in keeping with our current observations, IVRT has been shown to be prolonged in the prenatally growth‐restricted infant (Altın et al., 2012) with normalization by 5 years (Crispi et al., 2010). More in keeping with findings in the rat exposed to a hypoxic intrauterine environment, several clinical studies have shown evidence of increased aortic and/or arterial stiffness following exposure to placental insufficiency in the newborn (Akira & Yoshiyuki, 2006), young child (Crispi et al., 2012), preadolescent (Bradley et al., 2010), and young adult (Miles et al., 2011). Given the impact of long‐term increased arterial stiffness on the heart and cardiovascular health in general, it is possible increased arterial stiffness is a critical contributor to early myocardial and vascular remodeling and long‐term cardiovascular disease. There is currently no clinical study that has explored pathogenic mechanisms and pathophysiology that further explains the clinical pathology observed among affected children. There is also no clear link between early life LV functional changes and adult CVD which would be necessary to identify the highest risk patient population and evolve preventative and treatment strategies to optimize the long‐term outcome of affected patients.

### 4.3 Limitations

There are limitations of our study that warrant consideration. Our model examined fetal hypoxia derived through maternal hypoxia rather than primary placental insufficiency. This model is associated with increased maternal blood pressure and altered vascular reactivity and late gestation reduction in uterine arterial resistance (Aljunaidy, Morton, Cooke, & Davdige, 2016), a finding akin to observations in pregnant women at high altitude (Krampl et al., 2001) whose fetuses demonstrate IUGR (Jensen & Moore, 1997). While IUGR is present in this model and sheep models at high altitude (Parraguez et al., 2013), normal placental size and weight, not typical of primary placental insufficiency, has been demonstrated (Krampl et al., 2001). In fact, there is currently no animal model which fully reproduces the findings in human IUGR due to placental insufficiency in its entirety. In experimental investigations, IUGR has been induced through maternal malnutrition and low protein diet (Resnick, Morgane, Hasson, & Miller, 1982; Woods, Weeks, & Rasch, 2004), maternal iron deficiency (Gambling et al., 2002), hypoxic pregnancies (Bae et al., 2003; Botting et al., 2018, 2014; Li et al., 2003; Mortola et al., 1990; Tong et al., 2011; Xu et al., 2006), surgical ablation/ uterine vessel ligation (Morrison et al., 2008; Turner & Trudinger, 2009), and occlusion of the umbilical artery through direct ligation (Wadley et al., 2013) or embolization (Bubb et al., 2009). Unfortunately, models that perhaps better simulate the pathophysiology of placental insufficiency including surgical removal of portions of the uterus or umbilical placental embolization typically involve use of large animals such as sheep that are expensive and not as easily studied for the longer‐term impact of intrauterine exposures. Adding to the inadequacies of any animal model are species‐specific maturational elements which challenge their translation to human disease. For example, in larger animal models such as sheep, cardiac myocytes largely lose their capacity to proliferate before birth (Burrell et al., 2003; Jonker et al., 2007), whereas in rats, the cardiac myocytes undergo terminal differentiation midway through the first week after birth (Li et al., 1996). While terminal differentiation of cardiac myocytes in humans begins in the third trimester (Kim et al., 1992), by term, 90% of myocytes are still mononuclear suggesting an ongoing proliferative capacity and there remains a larger proportion of mononucleated cells up through the first year (Schmid & Pfitzer, 1985). In rats, with return to normoxic conditions at birth, there is an ongoing ability for cardiomyocyte proliferation that may impact the subsequent functional capacity of the heart and result in greater endowment of cardiomyocytes into adulthood. Whether in some way this relative immaturity of the early rat heart results in advantageous or deleterious remodeling of the myocardium, and how much it fully recapitulates the evolution of cardiovascular disease in humans remains uncertain. Still, our findings could be used to explore further pathogenic mechanisms responsible for early cardiovascular changes following fetal hypoxia exposure and novel interventional strategies early in life to prevent disease or risk factor progression.

One other limitation of our study was the small number of litters used, despite which, when examined accounting for litter, the majority of variables remained unchanged from our initial analysis. That only the LV lateral wall E’ at 2 weeks lost significance between groups accenting for the impact of the litter did not significantly impact our observations of progressive LV diastolic function changes. Although pup weight was no longer significantly smaller at day 1, the model used has been previously shown to be associated with variable IUGR. That we were unable to show a difference in pup size yet still demonstrated important differences in cardiac function suggests hypoxia alone, even in the absence of growth restriction contributes to important cardiovascular changes that impact longer‐term cardiovascular health.

## 5 CONCLUSION

In conclusion, prenatal exposure to hypoxia results in transient changes in heart rate and LV systolic function, more progressive changes in LV diastolic dysfunction from early developmental stages that suggest combined relaxation and compliance abnormalities, and increased aortic stiffness from the newborn period to preadult stages. Alterations in LV diastolic function could be explained at least in part by altered collagen deposition which may evolve secondary to increased aortic stiffness. Ongoing work is needed to link these early functional changes with later life cardiovascular disease and to the histological, cellular, and molecular changes that are the consequence of hypoxia exposure.

## CONFLICT OF INTEREST

None of the authors have a conflict of interest related to this work.
